# Screening and Efficacy Assessment of Deep Eutectic Solvents for Potential Application in Dental Cleaning

**DOI:** 10.3390/jfb17100488

**Published:** 2026-09-27

**Authors:** Yu Xia, Yong Cao, Subhan Mahmood, Sonia Mukwana, Shun Yao

**Affiliations:** School of Chemical Engineering, Sichuan University, Chengdu 610065, Chinasoniamukwana@stu.scu.edu.cn (S.M.)

**Keywords:** deep eutectic solvents, dental calculus, extrinsic stains, dental cleaning, antibacterial activity, safety

## Abstract

Background: Effective removal of tenacious calculus and extrinsic stains without oral irritation remains a major challenge in dental cleaning. Deep eutectic solvents (DESs) are tunable green solvents, but their potential for dental care is largely unstudied. This study screened DESs assembled from food-grade starting components and evaluated their cleaning efficacy, antibacterial activity and preliminary biosafety. Methods: Twelve DESs were synthesized and characterized. Their solubilization toward dental stain-related components was tested under stirring, ultrasonication and electro-stirring. The best-performing DESs were further assessed on natural dental calculus and stained bovine teeth, with comparison against commercial dental cleaners. Antibacterial effects and biosafety were also determined. Results: Hydrophilic low-pH DES-3 (maleic acid-xylitol) and DES-5 (lactic acid-citric acid-xylitol) efficiently dissolved calcium phosphate and pigments, while hydrophobic DES-12-P emulsion performed best for oily stains. Cleaning performance depended on DES concentration and agitation approaches. The optimized DES outperformed commercial products for complex mixed stains on bovine teeth with no enamel damage. It also displayed potent antibacterial effects against *Staphylococcus aureus* and *Escherichia coli*, with good biosafety in zebrafish tests. Conclusions: DESs exhibit favorable cleaning and antibacterial performance under the tested in vitro conditions, suggesting their potential as a candidate strategy for dental hygiene and oral care applications.

## 1. Introduction

Oral health is a crucial component of overall human health, and maintaining it relies heavily on daily cleaning and proper care. Currently, the most widely recommended methods by dental experts include brushing twice a day (in the morning and at night) and using dental floss to clean interdental spaces [1]. Although these measures have contributed positively to oral health, evolving lifestyles and increasingly diverse dietary habits continue to give rise to various oral health issues, as harmful substances progressively accumulate in the oral cavity, gradually eroding dental hard tissues [2]. Dental plaque is a soft, yellow, or white paste-like substance that forms on tooth surfaces. It consists of bacteria, food debris, and saliva. If not removed in a timely manner, plaque can mineralize and harden into dental calculus, which varies in color from yellowish-white to dark brown or black. Dental calculus is primarily composed of phosphate minerals, trace amounts of organic matter, and elements such as calcium and iron. It tends to accumulate on tooth surfaces, especially near the gingival margin where the tooth meets the gum tissue [3]. The irregular surface of dental calculus provides an ideal environment for bacterial colonization below the gumline, which can lead to gingivitis. Clinical symptoms of gingivitis include halitosis, redness, swelling, and bleeding of the gums. Once formed, calculus cannot be removed by regular brushing [4]. Furthermore, long-term consumption of beverages such as tea and coffee can cause persistent stains on the teeth, turning them yellow or black. This not only affects aesthetics but may also compromise dental health [5]. For daily home-care, a variety of toothpastes and mouth-rinsing products are commercially available to control plaque and reduce mild surface stains. Common active ingredients include fluoride, triclosan, cetylpyridinium chloride, and chlorhexidine gluconate. These formulations are effective for soft plaque and superficial fresh stains, yet they possess inherent drawbacks. They barely dissolve mature mineralized dental calculus and deposited mixed stains. Long-term application of these ingredients causes irritation and damage to the oral mucosa [6]. Consequently, there is a need to explore alternative cleaning candidates that can concurrently target mineralized deposits and extrinsic pigments, while lowering soft-tissue irritation risk.

DESs are a class of emerging green solvents formed by combining a hydrogen bond donor (HBD) and a hydrogen bond acceptor (HBA). These novel solvents remain liquid at room temperature and exhibit remarkable physicochemical properties and structural tunability [7]. Most importantly, DESs display outstanding solvation capacity, and their dissolution capability can be flexibly modulated by adjusting their composition and component ratios [8]. This allows them to be tailored for various solutes, including small organic molecules, inorganic salts, and biomass, and has therefore led to their widespread application in fields such as separation and extraction [9]. For instance, Jelinski et al. reported that a deep eutectic solvent composed of choline chloride and glycerol increased the solubility of curcumin by 12,000 times compared with an aqueous solution, while also significantly improving its stability and bioavailability [10]. Pedro et al. have demonstrated that DESs can improve the solubility, stability, and therapeutic efficacy of antibiotics such as ciprofloxacin, without inducing drug resistance or cytotoxicity [11]. However, despite these impressive advances in pharmaceutical and biomedical applications, the exploration of DESs for dental cleaning remains extremely limited. In particular, their potential in dissolving dental calculus, extrinsic stains, and other stubborn oral deposits has not been systematically explored, leaving a notable research gap for developing green and efficient dental hygiene products.

Dental calculus and extrinsic enamel stains form heterogeneous mineral–biofilm composites that hinder DES diffusion, restricting cleaning efficacy when DES is applied alone. Ultrasound, an established clinical dental technique, mechanically eliminates calcified deposits via vibration, cavitation, microjets and acoustic streaming [12]. Weak direct current can disturb biofilm structural integrity, weaken bacterial adhesion to hard tooth surfaces, and disrupt cross-linking between inorganic mineral fractions and organic biofilm components [13]. The combination of DES with ultrasound or weak electric current generates physicochemical synergy to overcome diffusion limitations within thick dental deposits [14]. Accordingly, this study presents a novel tooth-cleaning strategy integrating DESs with ultrasound and electric current. We systematically evaluate its performance in calculus and stain removal, characterize antibacterial activity and biosafety, and benchmark its efficacy against commercial oral cleaning formulations. This work may offer new insights for developing effective, mild and eco-friendly oral cleaning technologies.

## 2. Materials and Methods

### 2.1. Materials and Instruments

All reagents and solvents used in this study were of analytical grade or above. Specifically, menthol and lactic acid were obtained from Kelong Chemical Reagent Factory (Chengdu, China); xylitol, citric acid, maleic acid, and glycerol monostearate were supplied by Macklin Biochemical Co., Ltd. (Shanghai, China); and calcium phosphate, thymol, and malic acid were sourced from Aladdin Reagent Co., Ltd. (Shanghai, China). Caramel pigment was purchased from Bodanheng Bio-tech Co., Ltd. (Zhejiang, China). Baiyunshan oral care rinse (commercial dental cleaning water) was provided by Baiyunshan Yingkang Pharmaceutical Co., Ltd. (Guangxi, China). This ready-to-use formulation requires no dilution. Its primary ingredients include purified water, carbomer, sodium dihydrogen phosphate, and saccharin sodium, with a measured pH range of 5.0–7.5. Per the manufacturer’s instructions, the product is designed for daily oral cleaning and freshening. The recommended usage consists of 5–10 mL for gargling with a contact time of 10–30 s, followed by expectoration. The bacterial strains *Escherichia coli* (*E. coli*, CMCC44103) and *Staphylococcus aureus* (*S. aureus*, CMCC26003) were acquired from Shanghai Luwui Technology Co., Ltd. (Shanghai, China). Experimental deionized water was prepared using an ultrapure water (UP water) purification system (0.4 μm filter; Millipore Co., Ltd., Bedford, MA, USA).

The DESs were characterized using a Spectrum Two L1600300 Fourier transform infrared (FT-IR) spectrometer (PerkinElmer Inc., Waltham, MA, USA) and a JNM-ECZ400S/L1 nuclear magnetic resonance (NMR) spectrometer (JEOL Co., Ltd., Tokyo, Japan). The cohesion and viscosity of the DESs were measured with a TA.HTC-20 mass spectrometer (Baosheng Industrial Development Co., Ltd., Shanghai, China) and an NDJ-8S rotational viscometer (Hengping Instrument Factory, Shanghai, China), respectively. Absorbance was determined using a UV-VIS 2800 spectrophotometer (Shunyu Hengping Instrument Co., Ltd., Shanghai, China). The color and microstructure of the tooth surface were examined using a DS-220 colorimeter (Caipu Technology Co., Ltd., Hangzhou, China) and a JSM-7610F field emission scanning electron microscope (FESEM; JEOL Co., Ltd., Tokyo, Japan), respectively.

The tooth samples used in this study included dental calculus and bovine teeth. Dental calculus was collected from bovine teeth using a sterile curette [15], and the latter was bought from Alibaba online shopping platform. Moreover, those teeth with caries, restorations, cracks, fluorosis, or hypoplasia were strictly excluded [16,17]. All samples were rinsed three times with deionized water to remove residual saliva and debris, and then freeze-dried for subsequent analysis.

### 2.2. Synthesis and Characterization of DESs

As conventional food additives, menthol and xylitol were selected as HBAs, while citric acid, malic acid, lactic acid, maleic acid, and thymol served as HBDs. Molar ratios were selected via preliminary screening and optimization to produce stable, homogeneous, free-flowing deep eutectic liquids without solid precipitation at room temperature. The compositions and synthesis conditions of DESs are summarized in Table 1. The visual appearance of all synthesized DESs is depicted in Figure S1 (Supplementary Materials). As shown, all DESs formed colorless and transparent liquids, except for DES-12, which appeared as a pale-yellow transparent liquid. The chemical structures of these DESs were characterized by Fourier transform infrared (FT-IR) spectroscopy and ^1^H nuclear magnetic resonance (^1^H NMR) spectroscopy [7,18]. Owing to their hydrophobicity, DES-11 and DES-12 were emulsified to enable subsequent investigations. Accordingly, pseudo-ternary phase diagrams were constructed using the water titration method, employing PEG-40 hydrogenated castor oil and Tween 80 as surfactants, and UP water as the aqueous phase [19]. Detailed experimental methods are provided in the Supplementary Materials.

### 2.3. Dissolution of Calculus-Related Components by DESs

The dissolution efficacy of each system against key calculus-related components (calcium phosphate, tea stains, coffee stains, and oil stains) was evaluated using DES aqueous solutions or emulsions (see Table 2). All experiments were conducted under three auxiliary conditions as follows: (i) Mechanical stirring (simulating of actual teeth cleaning conditions with gargle)—stirred at 300 rpm for 10 min using a magnetic stir bar in a 25 °C water bath. (ii) Ultrasonication (simulating of actual ultrasound wave-assisted teeth cleaning conditions)—treated at 40 kHz for 10 min in a thermostatic ultrasonic cleaner at 25 °C with an effective acoustic power of 300 W. The ultrasonic tank had an internal volume of 0.8 L, and all sample containers were positioned in the central region of the ultrasonic bath. (iii) Electro-stirring (simulating of actual microcurrent-assisted teeth cleaning conditions)—stirred at 300 rpm for 10 min at 25 °C with electrodes immersed in the reaction system under an applied voltage of 6 V (four dry batteries connected in series).

#### 2.3.1. Dissolution of Calcium Phosphate by DESs

A 3 g sample of calcium phosphate (recorded as *W*_0_) was weighed, followed by the separate addition of 30 mL of each DES aqueous solution or emulsion. The mixture was stirred, then filtered, and rinsed three times with UP water. The collected solid residue was dried to constant weight and weighed to determine the mass of undissolved calcium phosphate (*W*_1_). The dissolution efficiency was then calculated using Equation (1) as follows [20]:

(1) $$R(\%)=\frac{W_{0}-W_{1}}{W_{0}}\times 100\%$$

where *R*, *W*_0_, and *W*_1_ represent the dissolution efficiency of calcium phosphate (%), the initial mass of calcium phosphate (g), and the residual mass after dissolution (g), respectively.

#### 2.3.2. Dissolution of Tea and Coffee Stains by DESs

Oolong tea leaves and coffee powders (10 g each) were separately added to 200 mL of UP water. The mixtures were heated with stirring for 30 min, cooled, and filtered to obtain the corresponding tea and coffee extracts. To prepare the composite-stained sample, 0.9 g of hydroxyapatite was sequentially treated with 30 mL of each extract for 3 min, with vortex mixing and centrifugation after each addition [21]. Subsequently, the stained samples were separately added into DES aqueous solutions or DES emulsions and subjected to agitation treatment under one of the three conditions described above. After treatment, the samples were filtered, rinsed three times with 20 mL of 50% ethanol aqueous solution, filtered again, and then dried. For the control group, UP water was used in place of the DES solutions, while all other procedures remained exactly the same. Each dried sample was mixed with 30 mL of 2 mol/L hydrochloric acid, vortexed, and centrifuged. The supernatant was collected, and its absorbance was measured at 350 nm on a UV-Vis spectrophotometer. All measurements were performed in triplicate. The cleaning efficiency, expressed as the *E* value, was calculated using the following equation [22]:

(2) $$E\;=\;\frac{A_{s}-A_{n}}{A_{n}}$$

where *E* denotes the cleaning efficiency for tea and coffee stains; *A_s_* and *A_n_* denote the absorbance values after cleaning with UP water and the DES sample, respectively.

#### 2.3.3. Dissolution of Oil Stains by DESs

A mixture of soybean oil (64 g), lard (8 g), tallow (8 g), and glycerol monostearate (2.4 g) was added to 250 mL of water, referring to the Chinese National Standard (GB/T 35833-2018) [23]. The mixture was stirred and heated at 70 °C until uniform, then cooled to 30 °C. 8 g of ammonia-free caramel color and 12 g of wheat flour were sequentially added, each followed by thorough stirring. The mixture was sealed with plastic wrap and aged at room temperature for 24 h to prepare the artificial oily soil. The stainless steel sheets (50 mm × 50 mm × 3 mm) were prepared, dried at 120 °C for 1 h, and then weighed to record the initial mass as *m*_0_. Artificial oily soil was applied to the center of each sheet using a brush, with the coating amount controlled to 0.24–0.26 g per sheet. The coated sheets were then dried at 200 °C for 10 min, followed by exposure to ultraviolet light for 3 h, and then aged in a desiccator for 24 h before being weighed again and recorded as *m*_1_. The soiled sheets were immersed in 150 g of DES aqueous solution or emulsion for 10 min, then gently rinsed by swaying in UP water for 30 s, and dried at 120 °C for 45 min. The final mass was recorded as *m*_2_. The oily soil removal efficiency, denoted as *f*, was calculated according to the following equation:

(3) $$f(\%)=\frac{m_{1}-m_{2}}{m_{1}-m_{0}}\times 100\%$$

where *f* represents the oil stain removal efficiency; *m*_1_, *m*_2_, and *m*_0_ denote the mass of the stainless-steel sheet before cleaning, after cleaning, and its original mass (g), respectively.

### 2.4. Cleaning Efficacy of DESs on Dental Calculus and Stains

#### 2.4.1. Cleaning Efficacy of DESs on Natural Dental Calculus

Based on the optimal DES for calcium phosphate dissolution selected in Section 2.3.1, three DES solutions at concentrations of 5%, 20%, and 40% were prepared for dental calculus dissolution experiments. The cleaned and dried natural dental calculus was weighed to obtain its initial mass *m*_0_. Subsequently, the calculus was added to 10 mL of the DES solution and treated using stirring, ultrasonication, and electro-stirring methods, respectively. After treatment, the sample was rinsed, dried, and re-weighed to determine the final mass *m*_1_. The mass of dental calculus removed per unit volume of DES aqueous solution was calculated using the following formula [24]:

(4) $$R=\frac{m_{0}-m_{1}}{V}$$

where *R* represents the mass of dental calculus cleaned per milliliter of DES aqueous solution (mg/mL); *m*_0_ and *m*_1_ are the initial and residual masses of calculus (mg); and *V* stands for the volume of the DES aqueous solution added (mL).

#### 2.4.2. Cleaning Efficacy of DESs on Bovine Teeth Stained with Tea or Coffee

Based on the DES identified in Section 2.3.2 as most effective against tea and coffee stains, solutions at 5%, 20%, and 40% concentrations were prepared for stain-cleaning tests. Bovine teeth were separately immersed in tea and coffee extracts for 24 h to induce staining (see Figure S2, Supplementary Materials). After rinsing and drying, the chromaticity of marked areas was measured before and after staining. The stained teeth were immersed in 30 mL of DES solution and treated separately by stirring, ultrasonication, or electro-stirring. The same individual stained bovine-tooth specimen was repeatedly measured at sequential time points (5, 10, 15, 20, 30, and 60 min): the tooth was taken out, dried for chromaticity measurement, and then returned to the DES solution to continue treatment for subsequent time-points. The color difference at each time point was calculated using the following formula [16]:

(5) $$\Delta E=\sqrt{\left[{\left(L_{t}-L_{1}\right)}^{2}+{\left(a_{t}-a_{1}\right)}^{2}{+\left(b_{t}-b_{1}\right)}^{2}\right]}$$

where ∆*E* is the total color difference, and *L*, *a*, *b* are the color-space coordinates (lightness, red–green, yellow–blue) with subscripts 1 and *t* denoting values before and after cleaning, respectively.

Subsequently, zero-order, first-order, Higuchi, and Ritger–Peppas kinetic models were applied to analyze the dissolution kinetics of colored substances from the tooth surface in DES solutions. The most appropriate model for describing the process was selected based on the fitting results.

#### 2.4.3. Cleaning Efficacy of DESs on Oil-Stained Bovine Teeth

Based on the DES identified in Section 2.3.3 as exhibiting optimal oil stain removal efficiency, aqueous solutions with mass fractions of 5%, 20%, and 40% were prepared. The viscosity, cohesiveness, and whiteness of each formulation were subsequently measured. Bovine teeth were first stained by immersion in the artificial oily soil (prepared as described in Section 2.3.3) for 24 h. The samples were then exposed to UV light for 3 h, gently rinsed with UP water, and dried. The stained teeth were placed in 30 mL of DES solution and subjected to agitation under stirring, ultrasonication, and electro-stirring conditions, respectively. The cleaning efficiency for oil stains was calculated according to the method described in Section 2.3.3.

### 2.5. Cleaning Efficacy of DESs on Mixed Stains on Bovine Teeth

Based on the screening results from Section 2.4, two DES aqueous solutions or emulsions in Table 2 demonstrating optimal dental stain removal efficacy were selected. Their cleaning performance on bovine teeth stained with mixed deposits was evaluated using a commercially available teeth-cleaning water as a control. Bovine teeth were separately immersed in tea extract, coffee extract, and artificial oily soil for 24 h. After rinsing and drying, the teeth were placed in 10 mL of DES solution and subjected to cleaning treatments via stirring, ultrasonication, and electro-stirring, respectively (as described in Section 2.3). The color difference values can be calculated according to the method described in Section 2.4.2. Subsequently, the microstructures of the tooth surfaces before staining, after staining, and after cleaning with DES solution were observed using a scanning electron microscope (SEM).

### 2.6. Antimicrobial Activity and Safety of DESs

#### 2.6.1. Antibacterial Activity Assessment

The Gram-negative bacterium *E. coli* and the Gram-positive bacterium *S. aureus* were selected as the test strains. A bacterial suspension was prepared following a previously described protocol [25]. The antibacterial activity of the samples was preliminarily evaluated using the agar diffusion method. Briefly, 100 μL of the bacterial suspension was evenly spread onto the surface of an agar plate. Sterile blank antibiotic discs (6 mm diameter) were then placed onto the inoculated agar. Subsequently, 10 μL of either the DES aqueous solution or the commercially available teeth-cleaning water was applied to each disc. The plates were incubated at 37 °C for 24 h. After incubation, the diameters of the inhibition zones were measured and recorded, with three replicates performed for each experimental group.

The morphological changes in *E. coli* and *S. aureus* before and after treatment with DES solution were observed using SEM. The dry bacterial samples were prepared using the following protocol: A 10 μL DES aqueous solution was added to suspensions of *E. coli* and *S. aureus* during their logarithmic growth phase (OD_600_ = 0.2–0.3), followed by incubation for 6–12 h. Subsequently, 1 mL of each bacterial suspension was centrifuged, and the resulting pellets were washed three times with 0.1 M phosphate-buffered saline (PBS, pH 7.4) to remove impurities. The bacterial pellets were then fixed with 2.5 wt% glutaraldehyde aqueous solution at 4 °C for 6 h, followed by dehydration through a graded ethanol series (30%, 50%, 70%, 80%, 90%, and 100% absolute ethanol) for 15 min at each concentration. The dehydrated pellets were subsequently treated sequentially with a 1:1 (*v*/*v*) mixture of isoamyl acetate and ethanol for 20 min, followed by pure isoamyl acetate for an additional 20 min. Finally, the samples were lyophilized under vacuum for 24 h to obtain dry bacterial powders.

#### 2.6.2. Application Safety Assessment

Zebrafish (*Danio rerio*) were used as the model organism in this study, with approval from the Animal Protection and Use Committee of Sichuan University (approval no. KS20240426; approval date: 26 September 2024). Fish of similar size (body length: 30 ± 5 mm; body weight: 0.3 ± 0.1 g) were selected from the same breeding tank and acclimated for at least 7 days under conditions matching those of the subsequent experiments. Feeding was discontinued 24 h prior to testing. All the fish used were clinically healthy during the two weeks preceding or throughout the experiment. A limit test was first performed by exposing ten zebrafish to 1 L of 100 mg/L DES solution for 96 h under constant environmental conditions, while no test sample was added to the control group. Experimental fish were considered dead when their gill covers were completely still for more than 1 min, unresponsive to touch and sunk, which would be quickly scooped out of the tested solution. According to China National Standard GB/T13267-1991 [26], if no mortality occurred during the limit test, no further acute toxicity testing was required, indicating with 99.9% confidence that the 96 h median lethal concentration (96 h-LC_50_) exceeded the tested concentration. If mortality was observed, a full acute toxicity study using a concentration series should be conducted, and any severe sublethal effects must be recorded.

## 3. Results

### 3.1. FT-IR and ^1^H NMR Spectral Analysis of DESs

Figure S3 (Supplementary Materials) displays the FT-IR spectra of DESs and their HBDs/HBAs. The spectra of the DESs show a superposition of the characteristic absorption peaks originating from their individual components, with no emerging new functional-group peaks. All DES samples exhibited broad and diffuse absorption bands in the range of 3200–3500 cm^−1^. Key ^1^H NMR signals of all DESs and their HBDs/HBAs are summarized in Table S1 (Supplementary Materials), and the ^1^H NMR spectra of DESs are shown in Figure S4 (Supplementary Materials). Partial free active hydrogen signals of raw monomers disappeared in DES systems. Hydroxyl proton signals originating from both HBDs and HBAs exhibited obvious downfield shifts with increased δ chemical shift values. Additional details are provided in the Supplementary Materials.

### 3.2. Emulsification and Pseudo-Ternary Phase Diagram Analysis

As DES-11 and DES-12 are hydrophobic, they cannot be directly applied for evaluating dental calculus cleaning performance in an aqueous system. Therefore, the two hydrophobic DESs were formulated as 40 wt% mixtures, with PEG-40 hydrogenated castor oil or Tween 80 as emulsifiers. As shown in Figure 1a, the DES/water binary system showed regular phase evolution with gradual addition of emulsifier. The system sequentially transformed from oil–water phase separation to viscous gel-like semi-solid, then to milky emulsion, and finally to transparent homogeneous microemulsion with increasing emulsifier dosage. Pseudo-ternary phase diagrams of four DES/emulsifier systems are presented in Figure 1b–e. The stable emulsification region of Tween 80 was markedly larger than that of PEG-40 hydrogenated castor oil for both DES-11 and DES-12.

### 3.3. Calcium Phosphate and Stain Cleaning Performance of DESs

Figure 2 illustrates the dissolution efficiency of 40 wt% DES solutions/emulsions against calcium phosphate, tea stain, coffee stain, and oil stain. The dissolution efficiency of all DES samples against calculus-related components was significantly higher than that of pure water. Hydrophilic DES aqueous solutions generally showed higher dissolution efficiency than hydrophobic DES emulsions for calcium phosphate, tea stains, and coffee stains. In contrast, hydrophobic DES emulsions exhibited superior performance in removing oily contaminants. Among them, DES-12-P, which contained the highest surfactant content, demonstrated the strongest oil removal efficacy. Within the same aqueous solution systems, dissolution efficiency correlated with pH. DES-3, with the lowest pH (pH 0.94), achieved a cleaning efficiency of approximately 0.8 against tea and coffee stains. However, dissolution efficiency did not strictly follow pH trends. DES-5 (pH 1.53) demonstrated the highest dissolution efficiency for calcium phosphate. Regarding agitation methods, the dissolution efficiency of calcium phosphate under ultrasonication was slightly higher than that under the other two treatment conditions. For hydroxyapatite stained with tea and coffee composite stains, ultrasonication was less effective than mechanical stirring.

### 3.4. Removal Performance of DESs Toward Natural Calculus and Stained Bovine Teeth

Based on the screening results from Section 3.3, the DES-5 aqueous solution (exhibiting the highest dissolution efficiency for calcium phosphate), the DES-3 aqueous solution (showing the best removal efficacy for tea and coffee stains), and the DES-12-P emulsion (demonstrating the optimal removal performance for oil stains) were selected for subsequent evaluation of their actual cleaning efficacy on natural dental calculus, tea-/coffee-stained bovine teeth, and oil-stained bovine teeth, respectively.

As shown in Figure 3a, the mass of dental calculus removed per milliliter of DES-5 solution increased gradually with DES-5 concentration (5%, 20%, and 40%). Among the three treatment methods, weak electro-assistance exhibited a moderate enhancing effect, while ultrasonication demonstrated the optimal dissolution efficiency. Figure 3b–d,f–h show the cleaning performance on tea-stained and coffee-stained bovine teeth. The whiteness of the teeth gradually increased, and the color difference (Δ*E*) rose accordingly with prolonged treatment time and higher DES concentration. After treating tea-stained bovine teeth with 40% DES-3 for 60 min, Δ*E* reached 35.35. Under identical treatment duration, coffee-stained teeth exhibited a Δ*E* of 44.30. Kinetic fitting analysis showed that tea stain removal more closely followed the Ritger–Peppas model, while coffee stain removal was more accurately described by the Higuchi model. Corresponding model parameters are detailed in Tables S2 and S3 (Supplementary Materials).

As shown in Figure 3e, the oil stain removal rate gradually increased with increasing DES concentration, reaching a maximum value of 46.62% at 40% concentration. Table S4 (Supplementary Materials) lists the composition and physical properties (cohesiveness, viscosity, and whiteness) of the DES emulsions at different concentrations, all of which increased with higher DES content.

### 3.5. Cleaning Performance of DESs Against Mixed Stains on Bovine Teeth

Using the optimally selected DES formulations (DES-3 and DES-12-P) and Baiyunshan oral care rinse (commercially available teeth-cleaning water) as a control, cleaning experiments were performed on bovine teeth sequentially stained with tea, coffee, and artificial oily soil to produce mixed stains. The cleaning efficacy of the DES-12-P emulsion was comparable to that of the commercial cleaning water, whereas the DES-3 aqueous solution exhibited significantly superior performance. Among the three cleaning methods, ultrasonication produced notably better results than both mechanical stirring and electro-stirring. Under ultrasonication, DES-3 achieved a maximum Δ*E* value near 25 (see Figure 4a) among all the results. Moreover, the Δ*E* values when the bovine teeth that were cleaned in three modes were re-stained by mixed substances are summarized in Figure 4b; the results indicate that DESs possessed stronger capacity to inhibit discoloration compared with the control group.

The surface morphologies of bovine teeth before staining, after staining with mixed deposits, and after cleaning with the DES solution and emulsion were characterized by SEM (see Figure 4c–f). Before staining, the enamel surface was smooth and flat, with no visible deposits. After staining, the enamel was completely covered by a large amount of massive agglomerates, forming cross-linked and stacked rounded protrusions. Following treatment with DES solution and emulsion, the composite deposit layer was largely stripped away, the pristine microstructure of the enamel was effectively restored, and surface residues were markedly reduced. Notably, no obvious acid-etch pits, grooves, cracks, or spot erosion were observed on the DES-cleaned enamel surface.

### 3.6. Antibacterial Activity and Bacterial Morphology Analysis of DESs

The antibacterial activities of DES-3, DES-5, and DES-12-P at two concentrations (5% and 40%) were evaluated using the zone of inhibition assay, with *E. coli* and *S. aureus* as the test strains (Figure 5a–d). The positive control showed almost no antibacterial effect, while all three DES samples exhibited favorable antibacterial efficacy. The inhibitory effect against *S. aureus* was markedly superior to that against *E. coli*. Notably, the 40% DES-12-P emulsion produced the largest inhibition zones, with diameters of 13.2 ± 0.3 mm and 24.1 ± 0.2 mm against *E. coli* and *S. aureus*, respectively—a difference of nearly two-fold (see Figure 5e). With increasing DES concentration, the antibacterial efficacy against both test strains was significantly enhanced, with a more pronounced improvement observed against *S. aureus*. For example, DES-5 showed an increase in inhibition zone diameter of approximately 50% against *E. coli*, while the increase reached about 155% against *S. aureus* (see Table S5, Supplementary Materials).

Morphological changes in bacteria before and after treatment with DES samples were observed using SEM, as shown in Figure 5f,g. Untreated controls showed *E. coli* with smooth rod-shaped surfaces and *S. aureus* as intact spherical cells, both exhibiting normal physiological morphologies. After treatment with the three DES samples, both bacterial types exhibited severe damage. Specifically, *E. coli* frequently displayed cell fragmentation, whereas *S. aureus* commonly showed extensive shrinkage and surface roughness.

### 3.7. Acute Toxicity Analysis of DESs

The safety of three 40% concentrated DES aqueous solutions and emulsions was evaluated using a zebrafish acute toxicity test. In the limit test, no mortality was observed in zebrafish exposed to DES-3 and DES-5 after 96 h (Figure 6a,b). The 96 h-LC_50_ values for DES-3 and DES-5 were determined to be greater than 100 mg/L (with 99% confidence). For DES-12-P, zebrafish exposed to a concentration of 100 mg/L rapidly exhibited abnormal behaviors, such as increased swimming speed and erratic darting, followed by a gradual loss of swimming ability, sinking to the bottom of the test container, and eventual death. Preliminary experiments revealed 100% mortality at 50 mg/L but 100% survival at 10 mg/L. Consequently, a test was conducted using a series of concentrations within the 10–50 mg/L range. The dose–effect relationship between the concentration of DES-12-P emulsion and the 96 h mortality rate of zebrafish was fitted using a linear regression method. As shown in Figure 6c, the fitted curve equation is y = 2.66667x − 19.68254 (*R*^2^ = 0.9323). Based on this curve, the calculated 96 h LC_50_ is 26.13 mg/L.

## 4. Discussion

### 4.1. Characterization of DESs

No new characteristic peaks were detected, indicating that no chemical reaction took place between the precursor components. The broad infrared absorption band observed in the range of 3200–3500 cm^−1^ demonstrates the formation of strong intermolecular hydrogen bonding networks among DES components, which are dominated by −O–H···O– interactions [17]. In the ^1^H NMR spectra, the disappearance of partial free active hydrogen and downfield shifts in hydroxyl proton chemical further demonstrated the reduction in electron cloud density around hydroxyl hydrogen atoms and the deshielding effect induced by hydrogen bonding. These spectral changes originate from the intrinsic hydrogen bond donor–acceptor interaction between HBDs/HBAs [27]. Collectively, the FT-IR and ^1^H NMR results confirmed the successful synthesis of the target DESs rather than simple physical mixing.

### 4.2. Emulsification and Phase Diagrams of Hydrophobic DESs

The phase evolution observed upon the gradual addition of emulsifier reflects the progressive stabilization of the interface. At the initial stage, the amount of emulsifier molecules is insufficient to fully cover the oil–water interface, forming only a discontinuous and incomplete interfacial adsorption film on the surface of the hydrophobic DES droplets. As the emulsifier dosage increases, surfactant molecules reduce the interfacial tension through interfacial adsorption and oriented alignment, causing large hydrophobic droplets to gradually break apart and disperse uniformly into the aqueous phase, ultimately forming a homogeneous and stable milky-white emulsion. When the emulsifier addition exceeds a critical concentration, the interfacial film becomes dense and complete, and the system finally transitions into a clear, transparent, and highly homogeneous microemulsion [28].

The emulsification region achieved with Tween 80 was significantly larger than that with PEG-40 hydrogenated castor oil, indicating that Tween 80 exhibited stronger solubilization capacity for both DES systems. Although the hydrophilic–lipophilic balance values of the two surfactants are similar, the C18:1 unsaturated chain of Tween 80 exhibits high flexibility due to its double bond. This structure allows deeper penetration into the hydrophobic network of the DES, thereby forming a dense and highly elastic interfacial film that effectively suppresses droplet coalescence and phase separation, significantly broadening the stable microemulsion region [29].

### 4.3. Dissolution of Calculus-Associated Constituents by DESs

Dental calculus is a common hard deposit on tooth surfaces, primarily formed by the deposition of inorganic salts, with calcium phosphate accounting for as much as 75.9% of its composition. Meanwhile, pigments (such as tea polyphenol oxidation products and caffeine derivatives) and acidic components (e.g., tannic acid, and chlorogenic acid) from tea and coffee stains can penetrate tooth enamel, leading to further pigmentation and promoting calculus accumulation [30]. Moreover, residual cooking oil stains serve as a nutrient source for anaerobic bacteria in the oral cavity. Volatile sulfur compounds and other byproducts of bacterial metabolism can directly contribute to oral malodor [31]. Collectively, these factors contribute to detrimental effects on dental health. Therefore, calcium phosphate, tea stains, coffee stains, and oil stains were selected as the research subjects.

The superior performance of DES samples compared to pure water is attributed to the unique hydrogen bond donor–acceptor network of DESs, which disrupts interactions between stain components and promotes dissolution and detachment of target substances [32]. Hydrophilic DES aqueous solutions generally outperformed hydrophobic DES emulsions in dissolving calcium phosphate, tea stains, and coffee stains. This superior performance arises from their low pH and high fluidity. The acidic environment disrupts the calcium phosphate lattice via protonation, promoting mineral dissociation, while the excellent fluidity enables rapid penetration into the stain–substrate interface, disrupting hydrogen bonding [33]. Additionally, the low viscosity of the fluid accelerates solute mass transfer and diffusion, thereby further enhancing the cleaning efficiency. In contrast, the hydrophobic DES emulsions exhibited superior performance in removing oily contaminants, with DES-12-P, which contained the highest surfactant content, demonstrating the strongest oil removal efficacy. This advantage is attributed to the amphiphilic structure of the surfactants: the molecules rapidly adsorb at the oil–substrate interface, with hydrophobic tails binding to the oil and hydrophilic heads forming hydrogen bonds with the aqueous phase. This reduces the interfacial tension and forms a stable emulsion, effectively removing oil stains through emulsification, solubilization, and dispersion [34].

Within the same aqueous solution systems, dissolution efficiency was found to correlate with pH. For example, DES-3, which had the lowest pH, exhibited the highest dissolution efficiency for tea and coffee stains. This can be attributed to the low-pH environment, which disrupts the ‘pigment-metal ion’ complexes through protonation, leading to the dissolution and decolorization of the pigments. Furthermore, DES molecules can form hydrogen bonds and dipole–dipole interactions with the polar groups of stain components, such as polyphenols, caffeine, and melanoidins, thereby promoting the dissolution of freed pigment molecules [35]. However, dissolution efficiency did not strictly follow pH trends. For instance, DES-5 demonstrated the highest dissolution efficiency for calcium phosphate. As an efficient calcium ion chelator, citric acid in DES-5 solution forms soluble complexes with calcium ions from calcium phosphate, thereby continuously shifting the dissolution equilibrium forward and significantly accelerating the dissociation and dissolution of the calcium phosphate crystals. These findings are consistent with previous literature reports [36].

Agitation methods also affect the cleaning efficacy of the DES system, with pronounced differences observed in their effectiveness against various types of stains. Ultrasonication exhibited a marginal advantage in calcium phosphate dissolution. This modest improvement is attributed to the cavitation effect, which disrupts the surface structure of the mineral and prolongs solvent–substrate contact time, thereby promoting more complete dissolution of calcium phosphate [37]. In contrast, ultrasonication was less effective in cleaning hydroxyapatite stained with tea and coffee composite stains. This is primarily because the pigments and calcium salts tend to form dense, agglomerated precipitates. Ultrasonic vibration struggles to break up these stable agglomerates and may even exacerbate particle compaction and aggregation, thereby reducing solvent penetration efficiency. By comparison, mechanical stirring effectively breaks up pigment–mineral composite agglomerates through continuous shearing and dispersion, increasing the contact area between DES and the stains and ultimately achieving efficient removal of extrinsic stains [38].

### 4.4. Cleaning Effect of DESs on Natural Dental Calculus and Stained Bovine Teeth

A single simulated substrate is insufficient to fully replicate the complex physiological microenvironment of the oral cavity. Natural dental calculus is a composite mineralized deposit formed by the intercrosslinking of calcium phosphate crystals, an organic matrix, and bacterial biofilms, with a complex composition that more closely represents the clinical situation [3]. Bovine teeth, as a well-established in vitro dental experimental model, possess a complete layered hard tissue structure comprising enamel and dentin, effectively replicating the authentic adhesion characteristics of extrinsic stains such as tea, coffee, and oil on tooth surfaces. Therefore, based on the screening results presented in Section 3.3, further comparative evaluations were conducted to assess the dissolution capability of these three optimized formulations against natural dental calculus, and their practical cleaning efficacy on stained bovine teeth, respectively.

The concentration-dependent increase in dental calculus dissolution rate with DES-5 is attributed to the concurrent increase in the concentrations of hydrogen bond donors/acceptors and calcium-chelating active sites. Consequently, interfacial penetration and mass transfer are enhanced, enabling more efficient disruption of both the crystalline lattice and the organic matrix of the dental calculus [39]. The moderate enhancing effect of weak electro-assistance is explained by the mild electric field dissociating hydrogen bonds within the DES system, increasing free ion concentration, and accelerating migration of calcium and phosphate ions from hydroxyapatite dissolution, thereby shifting the dissolution equilibrium forward [40]. In contrast, ultrasonication, through its cavitation effect, generates high temperatures, high pressures, shock waves, and micro-jets, and substantially enhances mass transfer, thereby demonstrating optimal dissolution efficiency.

The different kinetic models for tea and coffee stain removal revealed distinct cleaning mechanisms. The removal of tea stains more closely followed the Ritger–Peppas model, indicating that pigment release is primarily governed by DES-induced erosion of the stained matrix. DES molecules can rapidly penetrate into the interior of the deposition layer, where their erosive action disrupts the binding matrix between the pigment and the enamel, leading to the bulk detachment of pigment molecules. In contrast, the Higuchi model more accurately describes the cleaning process for coffee stains. This model indicates that the process is diffusion-controlled, in which pigment molecules gradually diffuse out of the matrix driven by the concentration gradient, with DES primarily acting as a penetrating extractant [41].

The increasing oil stain removal rate with DES concentration is highly correlated with the changes in the physical properties of the DES emulsion. As the DES content increased, the emulsification process became more uniform, and the emulsion structure tended to stabilize. Meanwhile, the higher viscosity and cohesiveness helped prolong the residence time of the emulsion on the tooth surface, thereby creating favorable conditions for the adsorption and emulsification removal of oil stains. This finding is consistent with previously reported results in the literature [42].

### 4.5. Removal Efficacy of DESs Against Mixed Stains on Bovine Teeth

The superior performance of DES-3 compared to both DES-12-P and the commercial cleaning water lies in its compositional characteristics. Maleic acid, as the HBD in DES-3, effectively disrupts both the hydrogen bonds and calcium coordination bonds between extrinsic pigments and enamel hydroxyapatite through its acidic dissociation properties and metal-chelating effects [43]. Notably, maleic acid has been demonstrated to effectively remove the smear layer on the inner wall of root canals and is suitable for the root canal preparation stage in endodontic treatment [44]. Meanwhile, xylitol, as the HBA in DES-3, is rich in polyhydroxyl structures and can form new hydrogen bonding networks with pigment molecules, thereby significantly enhancing the water solubility and dispersibility of the staining components [45]. At present, xylitol is commonly added to chewing gum as a sugar substitute. It can modulate dental plaque microbiota and reduce the abundance of cariogenic bacteria, while its well-known anti-cariogenic effect originates from the inhibition of carbohydrate metabolism of cariogenic bacteria [46].

The SEM observations indicate that polyphenols and macromolecular pigments from tea and coffee adsorb onto the enamel surface, while oil components act as an adhesive medium promoting aggregation and cross-linking of constituents, ultimately forming a continuous and compact composite deposit layer. The effective restoration of enamel microstructure after DES treatment, combined with the absence of acid-etch pits or cracks. These surface morphological results demonstrate the effective removal of composite stains. However, SEM only captures surface features and cannot exclude subsurface mineral loss or microstructural damage to enamel. For the primary objective of this study, namely assessing surface stain removal performance and screening for overt enamel surface corrosion, SEM characterization delivers sufficiently robust evidence to validate our main conclusions on stain cleaning. Additional complementary characterization techniques will be required for a comprehensive assessment of enamel integrity in future investigations.

### 4.6. Evaluation of Antibacterial Performance of DESs

Antibacterial activity is a core function of oral care products. Superior antibacterial performance can effectively inhibit the proliferation of oral bacteria, alleviate inflammation such as gingival swelling and pain, and thus play a vital role in maintaining oral health, improving the oral environment, and assisting in the treatment of oral diseases [47]. In this study, the common oral pathogens *E. coli* and *S. aureus* were selected as the test strains. *E. coli* is frequently associated with oral mucosal infections, while *S. aureus* is commonly isolated from periodontal abscesses and acute suppurative oral infections [48,49].

The superior inhibitory effect against *S. aureus* compared to *E. coli* is closely related to the distinct cell structural characteristics of the two bacteria. *S. aureus* lacks an outer membrane barrier, making it more susceptible to penetration by DES and subsequent disruption of its cellular structure. In contrast, the outer layer of *E. coli* is enveloped by an asymmetric outer membrane composed of lipopolysaccharides and phospholipids, which effectively impedes the penetration of DES and consequently reduces its antibacterial efficacy [50]. Additionally, the concentration-dependent enhancement in antibacterial activity observed herein is consistent with the literature [51]. Moreover, mouthwashes containing *Plectranthus amboinicus* (Lour.) Spreng extract and commercial mouthwashes exhibit inhibition zone diameters against *S. aureus* of 8.13–13.42 mm and 8.35 mm, respectively. In contrast, the DES samples tested in this study demonstrated markedly stronger antibacterial effects [52]. Notably, the commercial oral care rinse selected as positive control mainly consists of buffering agents, thickener, humectants and sweetener. Consequently, it shows negligible inhibitory zone in the agar disc-diffusion test. This result is consistent with its ingredient composition. It is not intended to serve as a strong antibacterial positive benchmark, and was included only as a representative marketed oral care commodity for parallel in vitro cleaning performance comparison.

The morphological damage to bacteria following DES treatment can be attributed to the synergistic antibacterial effects of the DES components. In DES-3, maleic acid inhibits bacterial glutamate decarboxylase and disrupts the cell membrane, while the included xylitol enhances this action by depleting bacterial energy and suppressing protective biofilm formation, resulting in a combined antibacterial effect [53,54]. Nonetheless, the strongly acidic pH of DES-3 (pH 0.94) and DES-5 (pH 1.53) may partially contribute to their antibacterial performance in this work. Organic acids in DES-5, including citric acid and lactic acid, can readily permeate the bacterial cytoplasmic membrane and dissociate in the more alkaline cytoplasm, causing intracellular acidification and accumulation of the acid anions, which further disrupts membrane integrity and cellular metabolism [55]. However, pH alone cannot fully explain the observed antibacterial potency. The near-neutral DES-12-P emulsion (pH 6.19) generated the largest inhibition zones against both *E. coli* and *S. aureus*, while the most acidic formulation, DES-3 (pH 0.94), did not deliver the strongest antibacterial activity. These findings demonstrate that the antibacterial performance of the DES systems is predominantly determined by their inherent bioactive components rather than acidity. For DES-12-P, thymol and menthol are both established antimicrobials: thymol directly disrupts cell membrane architecture, and menthol alters lipid packing within bacterial membranes, ultimately triggering the leakage of intracellular materials [56].

### 4.7. Safety Evaluation of DESs

According to OECD Test Guideline 203/EU Test Method C.1 [57], the 96 h-LC_50_ values for DES-3 and DES-5 being greater than 100 mg/L classify both as low-toxicity substances with a favorable safety profile, and no further toxicity testing was required. For DES-12-P, the calculated 96 h LC_50_ of 26.13 mg/L similarly classifies it as low-toxicity according to toxicity classification criteria. The toxicity observed in DES-12-P mainly originates from its component of menthol, which exhibits a certain degree of sensitive irritancy in fish. This result differs from that for humans. In a study in 2014, Japanese medaka, goldfish, and zebrafish were exposed to 0.5 mM menthol, resulting in 100% of the medaka and zebrafish, as well as 90% of the goldfish, entering a state of surgical anesthesia. At higher concentrations (such as 3.0 mM), fish will first exhibit rapid swimming and then enter an anesthetized state. It is obvious that humans perceive menthol mainly for its coolness, so it is widely used in chewing gum as well as oral cleaning products and is relatively safe. As a whole, both compounds in the tested DESs exhibit favorable biodegradability in the human body and are not prone to accumulation. Their biosafety has been confirmed by a large number of studies. They are also recognized to possess multiple physiologically beneficial effects, including tissue repair, local analgesia, and anti-allergic activity. Consequently, they are widely utilized across various sectors, such as pharmaceuticals, oral care, and the food industry [58,59].

## 5. Conclusions

In this work, twelve DESs synthesized from food-grade starting components were successfully prepared and structurally confirmed by FT-IR and NMR spectroscopy, while pseudo-ternary phase diagrams provided insight into the phase behavior of the hydrophobic DES systems. Initial screening of DES aqueous solutions and emulsions under varying agitation conditions—mechanical stirring, electro-stirring, and ultrasonication-identified three candidates (DES-3, DES-5, and DES-12-P) with marked dissolution capacity against calcium phosphate, tea/coffee stains, and oily deposits, respectively. Follow-up assessments using natural calculus and stained bovine teeth revealed a consistent dose-dependent improvement in cleaning efficacy, regardless of the agitation method applied. The optimized DES formulations exhibited superior performance in stain removal, deposit clearance, and anti-restaining capacity compared with the tested commercial oral rinse. Combined with their intrinsic antibacterial activity and low acute toxicity observed in zebrafish models, these results preliminarily demonstrate the promising potential of food-grade DESs as alternative auxiliary dental cleaning materials for oral hygiene intervention.

However, this study has several limitations. The strongly acidic nature of the DES formulations raises concerns regarding potential enamel demineralization. In addition, we did not perform cytotoxicity assays on human oral epithelial cells, cleaning efficacy tests on extracted human teeth, or evaluations of dentin desensitization efficacy; we also did not simulate real-world clinical application scenarios. Once adequate experimental infrastructure is in place and the requisite ethical approvals and clinical trial authorizations have been obtained, we will conduct in-depth investigations addressing these missing characterizations, alongside further formulation optimization and preclinical safety validation.

## Figures and Tables

**Figure 1:**
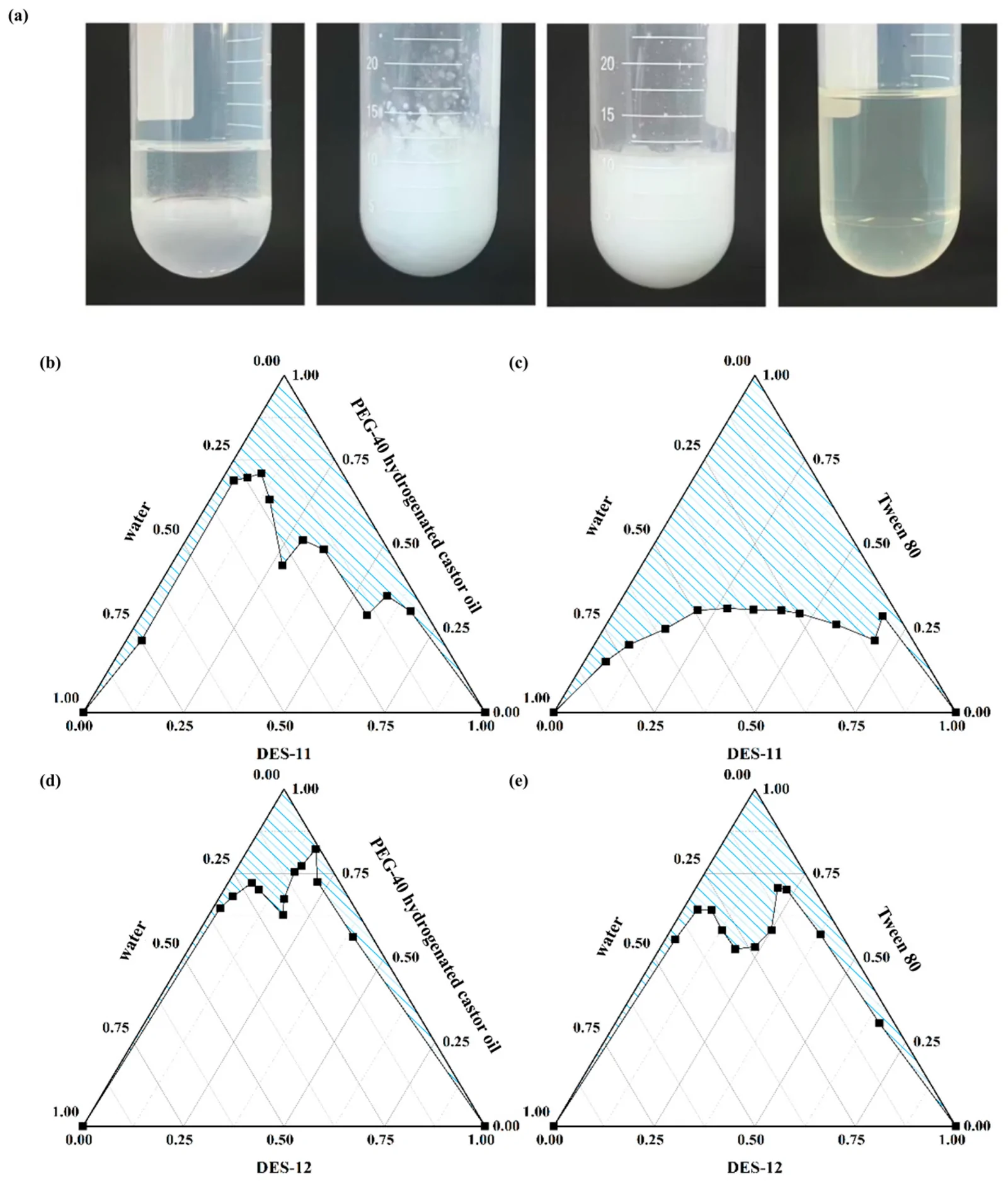
(**a**) Representative phase transition process of the DES/water system upon gradual addition of emulsifier (DES-11/Tween 80, 40% DES). Pseudo-ternary phase diagram of DES: (**b**) DES-11/PEG-40 hydrogenated castor oil; (**c**) DES-11/Tween 80; (**d**) DES-12/PEG-40 hydrogenated castor oil; (**e**) DES-12/Tween 80.

**Figure 2:**
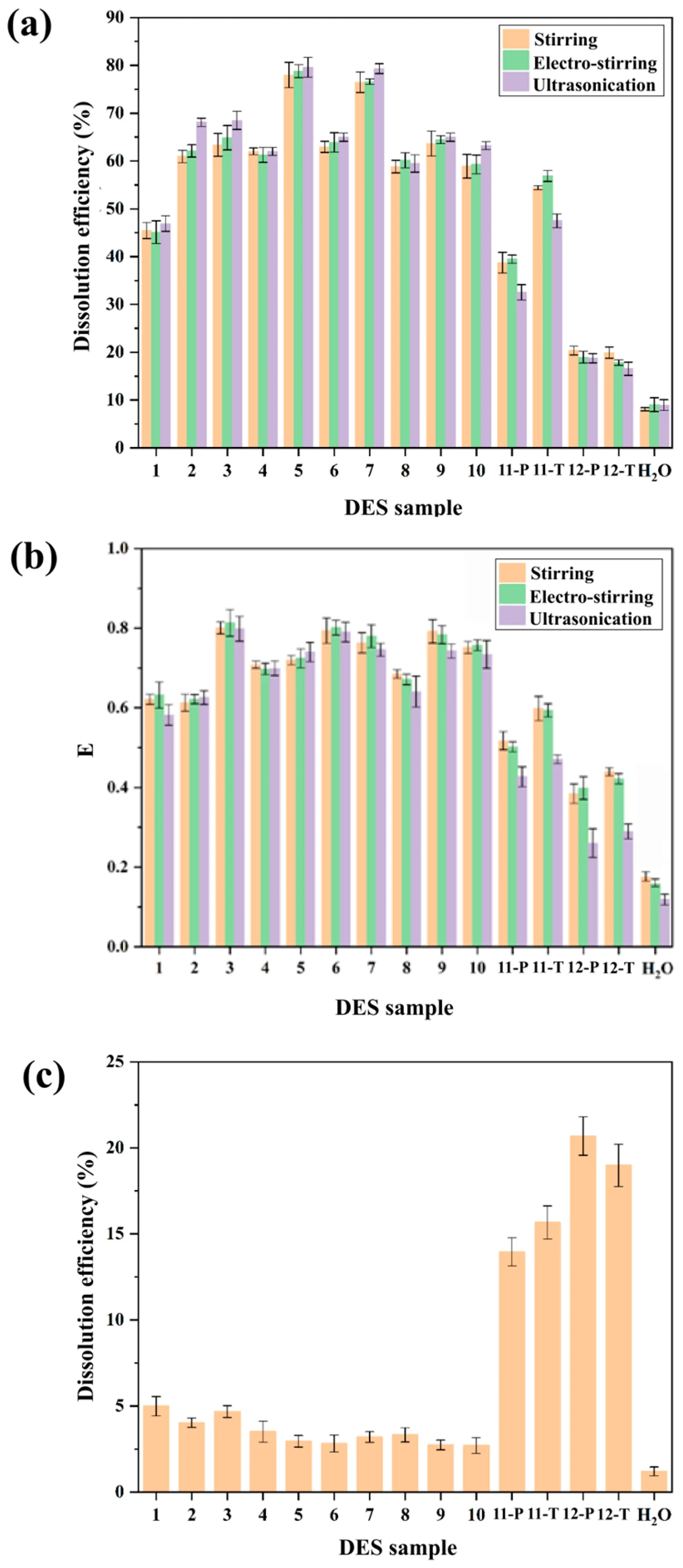
Dissolving efficiency of DESs for (**a**) calcium phosphate; (**b**) tea and coffee stains; and (**c**) oil stains.

**Figure 3:**
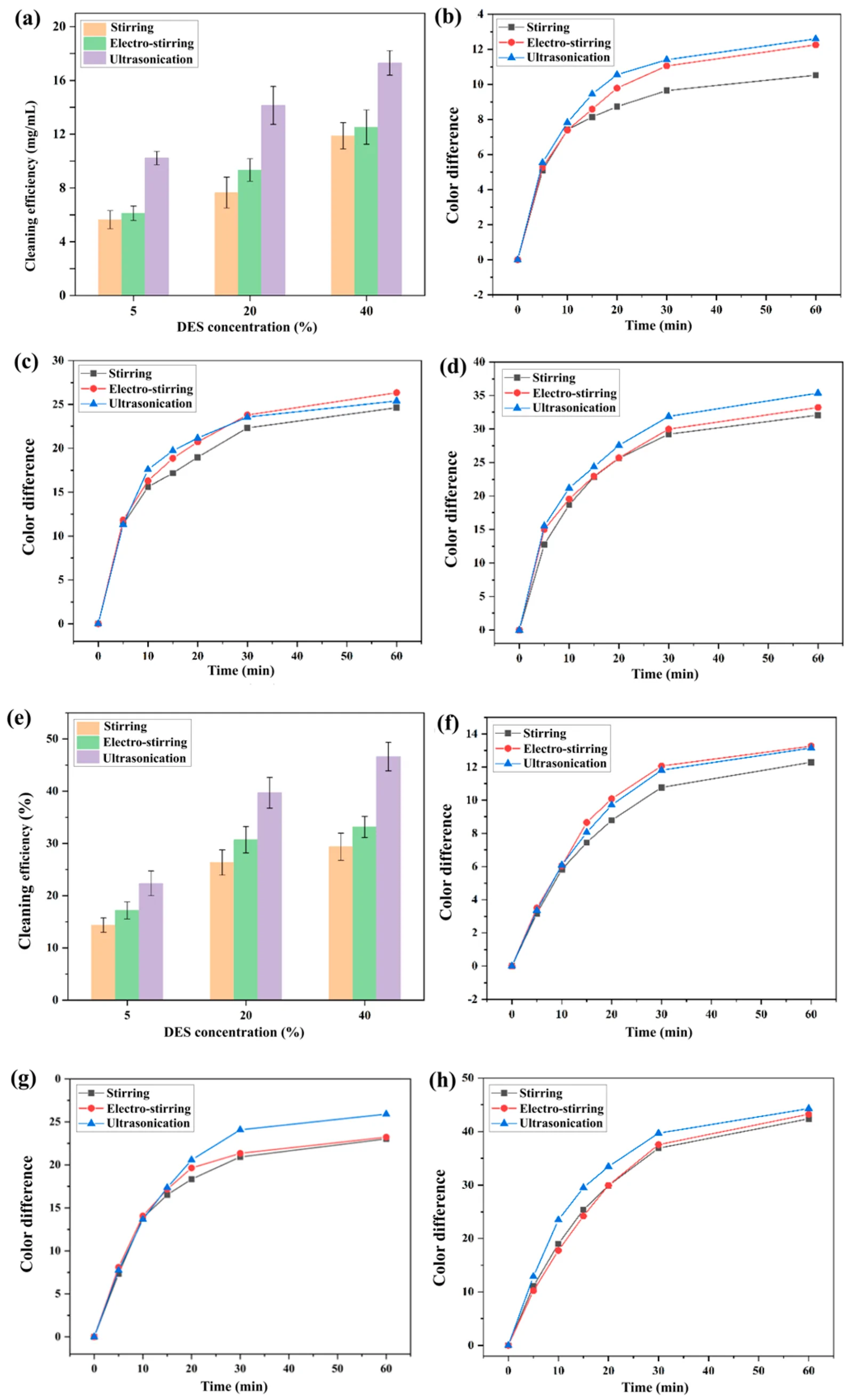
Cleaning performance of DESs on different substrates: (**a**) dental calculus with DES-5; (**b**–**d**) tea-stained bovine teeth with 5%, 20%, and 40% DES-3; (**e**) oil stains with DES-12-P; (**f**–**h**) coffee-stained bovine teeth with 5%, 20%, and 40% DES-3.

**Figure 4:**
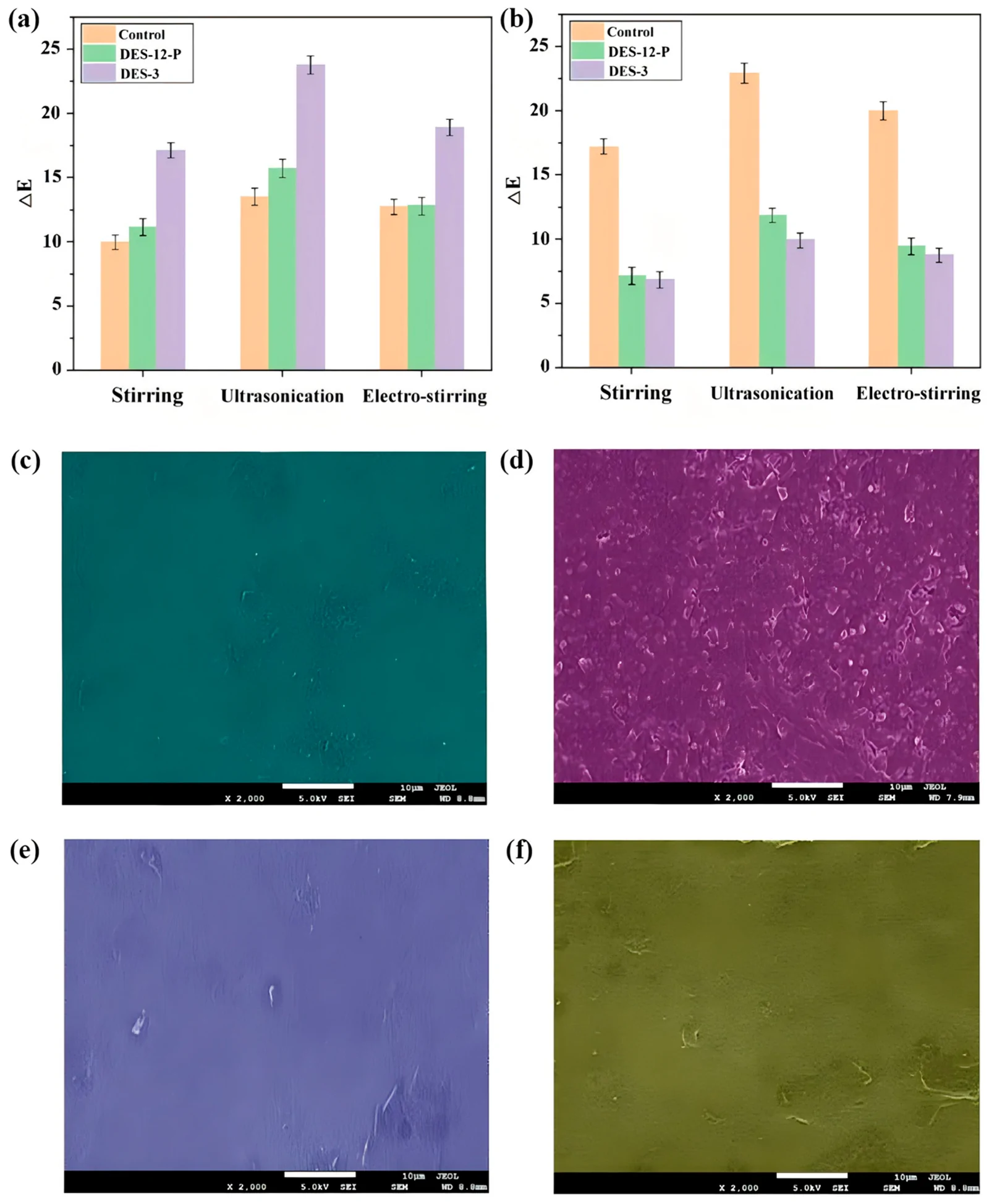
(**a**) DES cleaning effects of mixed stained bovine teeth and (**b**) changing results of re-stained bovine teeth. SEM images of bovine tooth surfaces at different stages. (**c**) Unstained surface; (**d**) surface after staining with mixed deposits; (**e**) Surface after cleaning with 40% DES-3 aqueous solution under ultrasonication (40 kHz, 10 min, 25 °C); (**f**) surface after cleaning with 40% DES-12-P emulsion under ultrasonication (40 kHz, 10 min, 25 °C).

**Figure 5:**
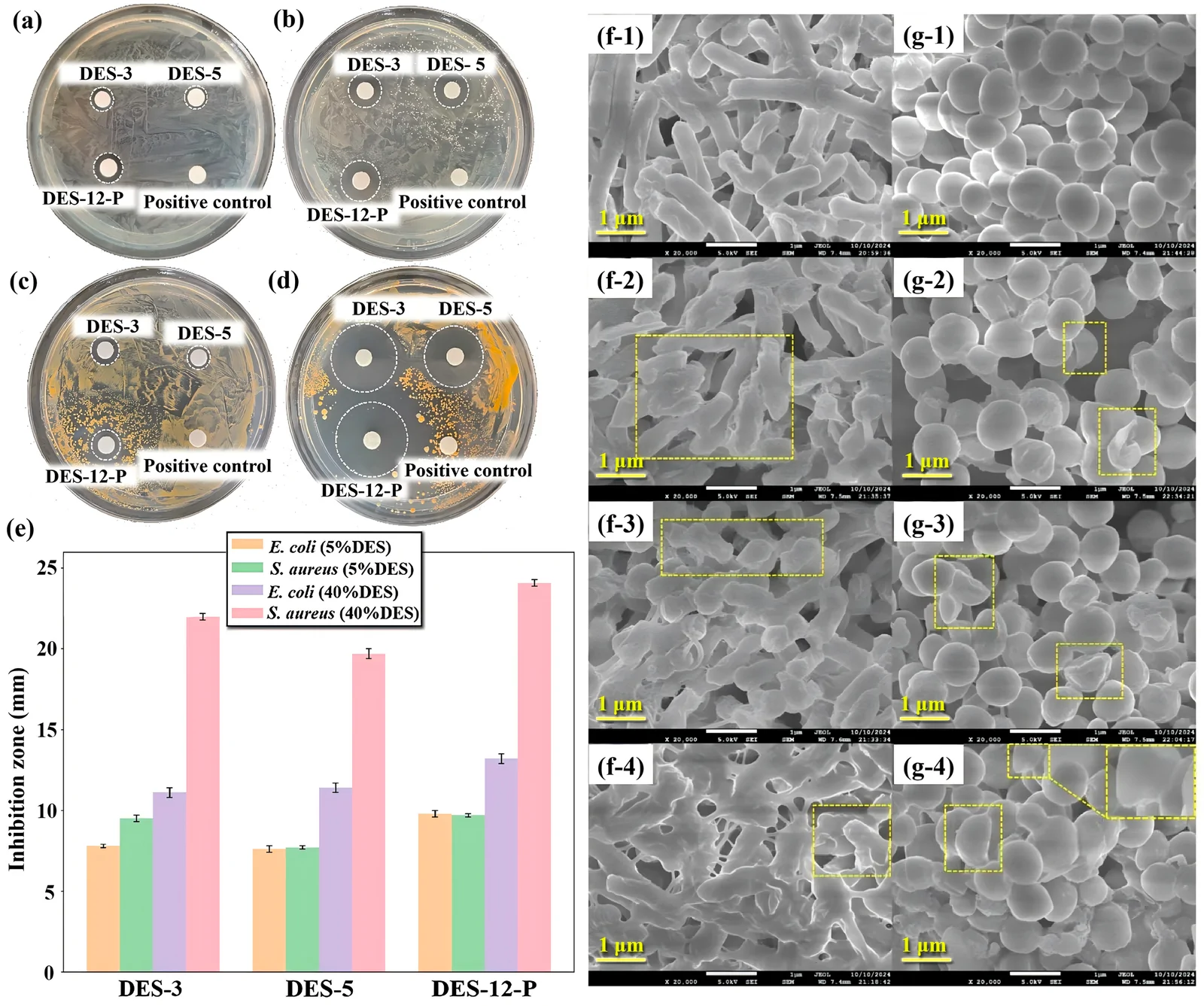
Antibacterial performance of three DES solutions and emulsions. Zone of inhibition test: antibacterial effects of 5% and 40% DES against (**a**,**b**) *E. coli* and (**c**,**d**) *S. aureus*. Each disc is labeled: DES-3, DES-5, DES-12-P, Positive control (commercial dental cleaning water). (**e**) Inhibition zone diameters at 5% and 40% DES against *E. coli* and *S. aureus*. SEM images showing morphological changes: untreated (**f-1**) *E. coli* and (**g-1**) *S. aureus*; (**f-2**,**g-2**) treated with 40% DES-3, (**f-3**,**g-3**) 40% DES-5, and (**f-4**,**g-4**) 40% DES-12-P.

**Figure 6:**
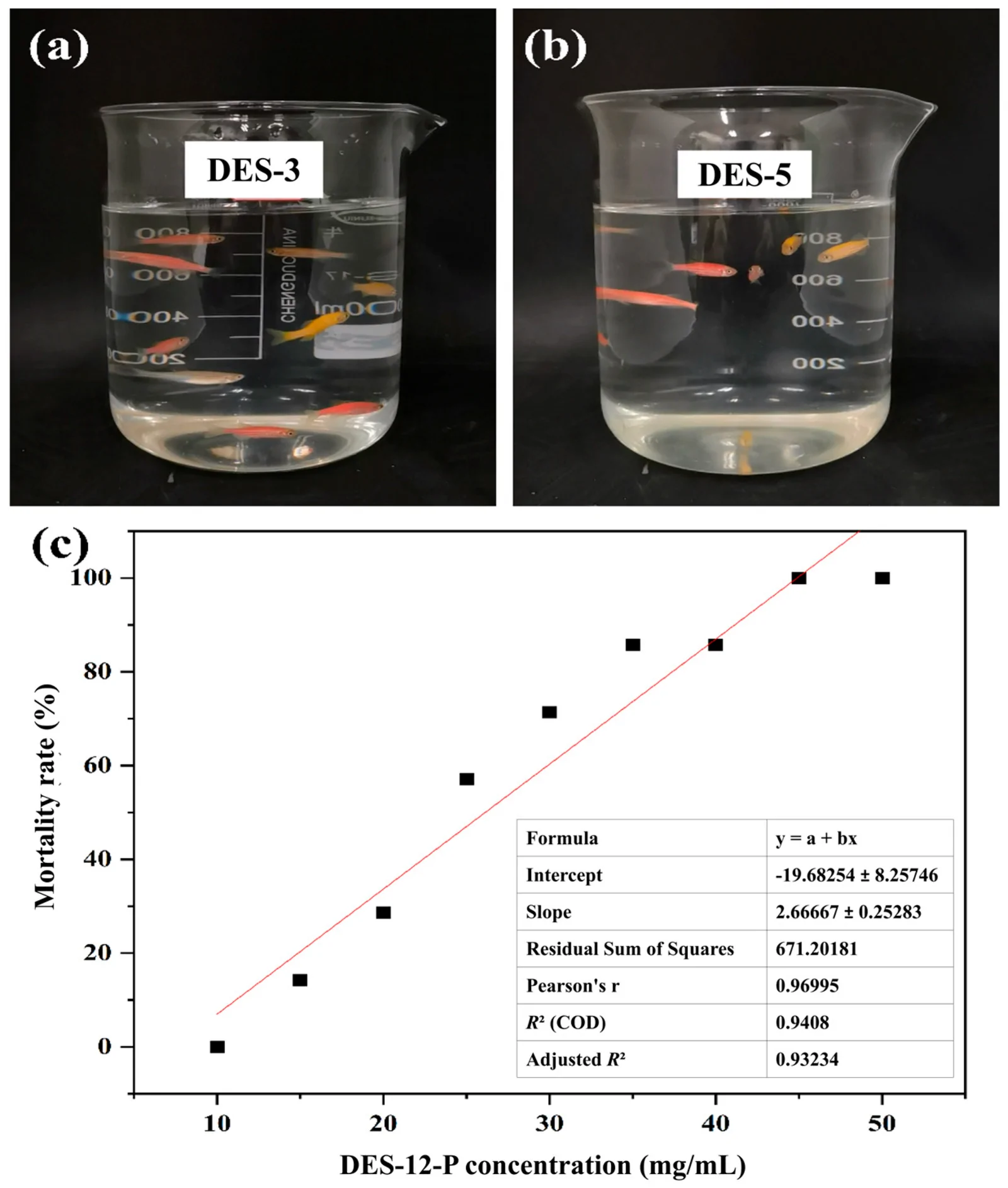
Images of zebrafish after 96 h of exposure to (**a**) DES-3 and (**b**) DES-5 aqueous solutions; (**c**) concentration–mortality fitting curve for the DES-12-P emulsion.

**Table 1:** Composition and synthesis conditions of DESs.

DESs	HBD		HBA	HBD/HBA	Reaction Temperature (°C)	Reaction Time (h)
DES-1	Lactic acid		Xylitol	1:1	80	2
DES-2	Citric acid			1:3	90	6
DES-3	Maleic acid			1:1	90	6
DES-4	Malic acid			1:1	90	6
DES-5	Lactic acid	Citric acid		1:1:2	80	2
DES-6	Lactic acid	Maleic acid		1:1:1	80	2
DES-7	Lactic acid	Malic acid		1:1:1	80	2
DES-8	Citric acid	Malic acid		1:1:3	90	6
DES-9	Maleic acid	Malic acid		1:1:2	90	6
DES-10	Maleic acid	Citric acid		1:1:4	90	6
DES-11	Lactic acid		Menthol	1:1	60	2
DES-12	Thymol			1:1	60	2

**Table 2:** Composition ratio of DES aqueous solutions or emulsions.

Code	Based DES	Surfactant	Aqueous Phase	DES/Surfactant/Water	pH
DES-1	DES-1	/	UP water	2:0:3	1.8
DES-2	DES-2	/			1.67
DES-3	DES-3	/			0.94
DES-4	DES-4	/			1.49
DES-5	DES-5	/			1.53
DES-6	DES-6	/			0.94
DES-7	DES-7	/			1.47
DES-8	DES-8	/			1.52
DES-9	DES-9	/			1.03
DES-10	DES-10	/			1.19
DES-11-P	DES-11	PEG-40 hydrogenated castor oil		4:1:5	1.9
DES-11-T	DES-11	Tween 80		4:1.5:4.5	1.77
DES-12-P	DES-12	PEG-40 hydrogenated castor oil		4:2:4	6.19
DES-12-T	DES-12	Tween 80		4:0.8:5.2	6.82

## Data Availability

All data supporting the findings of this study are contained within the manuscript and its Supplementary Materials.

## Supplementary Materials

The following supporting information can be downloaded at https://www.mdpi.com/article/10.3390/jfb17100488/s1. S1: Structural and emulsifying properties measurement of DESs; S2: Spectral analysis of DESs. Figure S1: Synthetic appearance of DESs; Figure S2: Marked and stained bovine teeth sample. Figure S3: Infrared spectra of DESs and their HBDs/HBAs (KBr disc). Figure S4: ^1^H NMR spectra of DES and their HBDs/HBAs (400 MHz, DMSO-*d*_6_). Table S1: Key ^1^H NMR signals of DESs and their components (400 MHz, DMSO-*d*_6_). Table S2: Fitting results of the two models for DES cleaning of tea-stained bovine teeth. Table S3: Fitting results of the two models for DES cleaning of coffee-stained bovine teeth. Table S4: Parameters of DES-12 emulsion. Table S5: Inhibition zone sizes of 5% and 40% DES against *E. coli* and *S. aureus*.

## Abbreviations

The following abbreviations are used in this manuscript:

DESs	Deep eutectic solvents
HBD	Hydrogen bond donors
HBA	Hydrogen bond acceptors
*E. coli*	*Escherichia coli*
*S. aureus*	*Staphylococcus aureus*
FT-IR	Fourier transform infrared
NMR	Nuclear magnetic resonance
FESEM	Field emission scanning electron microscope
96 h-LC_50_	96 h median lethal concentration
