# Supplementary material for: Screening and Efficacy Assessment of Deep Eutectic Solvents for Potential Application in Dental Cleaning
Source: J Funct Biomater. 2026 Sep 27;17(10):488. doi: 10.3390/jfb17100488 (PMC13705945; doi:10.3390/jfb17100488)
Supplement: Supplementary file 1 [file jfb-17-00488-s001.zip › jfb-4541929-supplementary.pdf]

Supplemental Information

Screening and efficacy assessment of deep eutectic solvents for potential application in dental cleaning

Yu Xia, Yong Cao, Subhan Mahmood, Sonia Mukwana, Shun Yao*

School of Chemical Engineering, Sichuan University, Chengdu 610065, China

2 sections, 5 tables, 4 figures, 3 references, 17 pages

Journal name : Journal of Functional Biomaterials

***Corresponding author.**

Prof. Dr. Shun Yao

E-mail: Cusack@scu.edu.cn(S. Yao). Tel.: +86-028-85405221; Fax: +86-028-85405221

Supplemental Information

Table of contents

Structural and emulsifying properties measurement of DESs P3

Spectral analysis of DESs P4

Table S1 P8

Table S2 P9

Table S3 P10

Table S4 P11

Table S5 P12

Fig. S1 P13

Fig. S2 P14

Fig. S3 P15

Fig. S4 P16

References P17

Supplemental Information

S1. Structural and emulsifying properties measurement of DESs

As shown in Fig. S1, all the prepared DESs were colorless and transparent clear liquids with a certain viscosity, except for DES-12.

S1.1. FT-IR analysis

For FT-IR analysis, the synthesized DES samples were mixed with dried KBr and pressed into pellets. Spectra were then recorded over a wavenumber range of 4000 to 450 cm^{-1} with a resolution of 4 cm^{-1}[1].

S1.2. 1H NMR analysis

The ^1H NMR spectra were acquired under the following conditions: 10 mg of sample was dissolved in 0.5 mL of DMSO-d_6. Spectra were recorded with 32 scans, a spectral width of 10,000 Hz, an acquisition time of 3.17 s, a relaxation delay of 1 s, and an operating frequency of 400 MHz[2].

S1.3. Phase Diagram of Hydrophobic DESs

The pseudo-ternary phase diagrams were constructed as follows: DES and UP water were prepared at specific mass ratios (1:20, 1:9, 2:8, 3:7, 4:6, 5:5, 6:4, 7:3, 8:2, 9:1, and 20:1). Each mixture was stirred continuously at room temperature, then titrated with the surfactant at a rate of 1 mL/min until the system transitioned from a cloudy, white emulsion to a transparent solution[3]. The volume of surfactant added at this transition point was recorded. Finally, the phase diagrams were plotted based on the mass fractions of DES, UP water, and surfactants.

Supplemental Information

S2. Spectral analysis of DESs

S2.1. FT-IR analysis

Taking DES-1 (lactic acid-xylitol) as an example, the FT-IR spectra of its components were analyzed. In the spectrum of lactic acid, characteristic peaks are observed at 3490 cm^{-1} (O-H stretching), 2989 and 2944 cm^{-1} (symmetric and asymmetric C-H stretching), 1731 cm^{-1} (C=O stretching), 1454 cm^{-1} (C-H bending), 1375 cm^{-1} (methyl C-H symmetric bending), 1223 cm^{-1} (C-O stretching), and 919 cm^{-1} (out-of-plane carboxylic O-H bending). In the spectrum of xylitol, which contains multiple hydroxyl groups, the hydroxyl group stretching appears in the 3200-3500 cm^{-1} region, the hydroxyl group in-plane bending occurs between 1400 and 1480 cm^{-1}, strong C-O(H) stretching is observed at 1025-1200 cm^{-1}, and C-H symmetric and antisymmetric stretching appears near 2900 cm^{-1}. The FT-IR spectrum of DES-1 exhibits a superposition of the characteristic peaks from both monomers, with no new peaks emerging, indicating that no chemical reaction took place between the components. Notably, a broad and diffuse absorption band appears in the 3200-3500 cm^{-1} region, confirming the presence of intermolecular hydrogen-bonding interactions in the DES system.

Taking DES-12 (thymol-menthol) as an example, the FT-IR spectra of its components were analyzed. In the thymol spectrum, characteristic peaks appear at 3284 cm^{-1} (O-H stretching), 2923 and 2851 cm^{-1} (C–H stretching of the CH_3 groups and the isopropyl C–H), 1622 cm^{-1} (aromatic C=C skeletal stretching of the benzene ring), 1400 cm^{-1} (C-H bending), and 1038 cm^{-1} (aromatic C–H in-plane bending). In the menthol spectrum, the O-H stretching vibration is observed at 3257 cm^{-1}, while C-H symmetric and antisymmetric stretching vibrations appear around 2900 cm^{-1}. The FT-IR spectrum of DES-12 reflects a superposition of the characteristic peaks from

both monomers, with no new peaks emerging, indicating the absence of a chemical reaction between the components. Notably, a broad and diffuse absorption band appears at 3375 cm^{-1} in the DES spectrum, with the O-H stretching vibration exhibiting a blue shift relative to that of pure thymol and menthol. The merging and broadening of the O-H band indicate that the hydroxyl groups of thymol and menthol participate in a common intermolecular hydrogen-bonding network within the DES, supporting the formation of intermolecular hydrogen bonds rather than chemical reactions between the components.

The FT-IR spectra of all the above DESs and their components are shown in Fig. S3.

S2.2. ^{1}H NMR analysis

The HBAs and HBDs used in DESs and their corresponding ^{1}H-NMR data are listed below:

For lactic acid: ^{1}H NMR (400 MHz, DMSO-d_6) δ 4.03 (q, J = 7.0 Hz, 1H), 1.22 (d, J = 7.0 Hz, 2H).

For citric acid: ^{1}H NMR (400 MHz, DMSO-d_6) δ 12.39 (s, 2H), 2.82-2.62 (m, 4H).

For malic acid: ^{1}H NMR (400 MHz, DMSO-d_6) δ 12.37 (s, 2H), 4.25 (dd, J = 7.8, 4.8 Hz, 1H), 2.60 (dd, J = 15.6, 4.8 Hz, 1H), 2.43 (dd, J = 15.7, 7.9 Hz, 1H).

For maleic acid: ^{1}H NMR (400 MHz, DMSO-d_6) δ 6.30 (s, 1H), 6.27 (s, 1H).

For xylitol: ^{1}H NMR (400 MHz, DMSO-d_6) δ 3.51 (td, J = 5.9, 3.3 Hz, 2H), 3.49-3.42 (m, 2H), 3.36 (tt, J = 10.7, 6.2 Hz, 2H).

For thymol: ^{1}H NMR (400 MHz, DMSO-d_6) δ 9.05 (s, 1H), 6.95 (d, J = 8.1 Hz, 1H), 6.58-6.52 (m, 1H), 3.13 (p, J = 6.7 Hz, 1H), 2.16 (d, J = 1.7 Hz, 3H), 1.11 (d, J = 7.0 Hz, 6H).

For menthol: ^{1}H NMR (400 MHz, DMSO-d_6) δ 4.29 (s, 1H), 3.15 (td, J = 10.2,

4.3 Hz, 1H), 2.18 (pd, J = 7.0, 2.5 Hz, 1H), 1.81 (dtd, J = 12.2, 3.8, 2.1 Hz, 1H), 1.59 (dp, J = 12.3, 2.9 Hz, 1H), 1.50 (dq, J = 12.3, 2.7 Hz, 1H), 1.33 (tdq, J = 12.8, 6.5, 3.3 Hz, 1H), 0.86-0.84 (m, 6H), 0.72 (d, J = 7.0 Hz, 3H).

For DES-1 (xylitol-lactate): ^{1}H NMR (400 MHz, DMSO-*d6*) δ 4.04 (q, J = 6.9 Hz, 1H), 3.52 (td, J = 5.7, 3.7 Hz, 2H), 3.48-3.41 (m, 2H), 3.35 (dd, J = 10.8, 6.0 Hz, 2H), 1.23 (d, J = 6.9 Hz, 2H).

For DES-2 (citric acid-xylitol): ^{1}H NMR (400 MHz, DMSO-*d6*) δ 12.40 (s, 2H), 4.32 (s, 2H), 3.54 (dp, J = 15.0, 5.2 Hz, 4H), 3.45 (dq, J = 10.4, 5.4 Hz, 4H), 3.39-3.24 (m, 4H), 2.96-2.62 (m, 4H).

For DES-3 (maleic acid-xylitol): ^{1}H NMR (400 MHz, DMSO-*d6*) δ 6.42-6.34 (m, 1H), 6.33-6.23 (m, 1H), 4.13 (q, J = 9.9 Hz, 2H), 3.57-3.49 (m, 2H), 3.46-3.42 (m, 2H), 3.37 (p, J = 5.6 Hz, 2H).

For DES-4 (malic acid-xylitol): ^{1}H NMR (400 MHz, DMSO-*d6*) δ 12.37 (s, 2H), 4.42-4.33 (m, 2H), 4.26 (dd, J = 7.8, 4.8 Hz, 1H), 3.52 (td, J = 5.7, 3.7 Hz, 2H), 3.48-3.41 (m, 2H), 3.35 (dd, J = 10.8, 6.0 Hz, 2H), 2.59 (d, J = 4.9 Hz, 1H), 2.44 (dd, J = 15.7, 7.8 Hz, 1H).

For DES-5 (lactic acid-citric acid-xylitol): ^{1}H NMR (400 MHz, DMSO-*d6*) δ 12.51 (s, 2H), 4.04 (s, 1H), 3.54-3.49 (m, 2H), 3.48-3.41 (m, 2H), 3.34 (dd, J = 10.9, 6.1 Hz, 2H), 2.78-2.61 (m, 4H), 1.29-1.20 (m, 2H).

For DES-6 (lactic acid-maleic acid-xylitol): ^{1}H NMR (400 MHz, DMSO-*d6*) δ 6.36 (d, J = 8.5 Hz, 1H), 6.26 (s, 2H), 4.04 (q, J = 6.9 Hz, 1H), 3.54-3.50 (m, 1H), 3.44 (ddd, J = 7.5, 5.4, 3.4 Hz, 3H), 3.35 (dd, J = 10.8, 6.0 Hz, 1H), 1.24 (dd, J = 9.0, 7.0 Hz, 3H).

For DES-7 (lactic acid-malic acid-xylitol): ^{1}H NMR (400 MHz, DMSO-*d6*) δ 12.49 (s, 2H), 4.25 (dd, J = 7.7, 4.8 Hz, 1H), 3.55-3.50 (m, 2H), 3.44 (dq, J = 10.3, 4.8 Hz, 2H), 3.36 (dd, J = 10.4, 4.5 Hz, 2H), 2.60 (dd, J = 15.7, 4.7 Hz, 1H), 2.47-2.40 (m,

1H), 1.25 (dd, J = 17.6, 10.9 Hz, 2H).

For DES-8 (citric acid-malic acid-xylitol): ^{1}H NMR (400 MHz, DMSO-d_6) δ 12.37 (s, 4H), 4.25 (dd, J = 7.7, 4.8 Hz, 1H), 3.52 (td, J = 5.8, 3.7 Hz, 6H), 3.43 (tt, J = 5.7, 2.1 Hz, 6H), 3.38-3.34 (m, 6H), 2.80-2.61 (m, 4H), 2.45 (dd, J = 7.9, 2.8 Hz, 1H).

For DES-9 (maleic acid-malic acid-xylitol): ^{1}H NMR (400 MHz, DMSO-d_6) δ 6.37 (s, 1H), 6.35 (s, 1H), 4.36 (ddd, J = 8.2, 4.5, 1.8 Hz, 4H), 4.26 (dd, J = 7.8, 4.8 Hz, 1H), 3.54 (dd, J = 5.4, 1.7 Hz, 4H), 3.46 (s, 4H), 3.39-3.35 (m, 4H), 2.61 (dd, J = 15.6, 4.8 Hz, 1H), 2.44 (dd, J = 15.8, 7.8 Hz, 1H).

For DES-10 (maleic acid-citric acid-xylitol): ^{1}H NMR (400 MHz, DMSO-d_6) δ 6.35 (s, 1H), 6.25 (s, 1H), 3.54-3.49 (m, 2H), 3.43 (q, J = 7.0 Hz, 2H), 3.34 (dd, J = 10.8, 6.0 Hz, 2H), 2.79-2.61 (m, 4H).

For DES-11 (lactic acid-menthol): ^{1}H NMR (400 MHz, DMSO-d_6) δ 4.32 (s, 1H), 4.06-4.00 (m, 1H), 3.15 (s, 1H), 2.17 (ddt, J = 11.3, 7.0, 3.7 Hz, 1H), 1.84-1.78 (m, 1H), 1.62-1.56 (m, 1H), 1.50 (dt, J = 12.4, 2.8 Hz, 1H), 1.35-1.30 (m, 1H), 1.25-1.21 (m, 2H), 0.86-0.83 (m, 6H), 0.73-0.70 (m, 3H).

For DES-12 (thymol-menthol): ^{1}H NMR (400 MHz, DMSO-d_6) δ 9.07 (s, 1H), 6.94 (d, J = 7.7 Hz, 1H), 6.60-6.50 (m, 1H), 4.30 (d, J = 5.5 Hz, 1H), 3.19-3.09 (m, 2H), 2.16 (s, 3H), 1.86-1.78 (m, 1H), 1.63-1.55 (m, 1H), 1.50 (dp, J = 12.3, 3.1 Hz, 1H), 1.38-1.27 (m, 1H), 1.11 (d, J = 6.9 Hz, 6H), 0.89-0.83 (m, 6H), 0.72 (d, J = 6.8 Hz, 3H).

The ^{1}H NMR spectra of DESs and their components are shown in Fig. S4.

Supplemental Information

Table S1. Key ^{1}H NMR signals of DESs and their components (400 MHz, DMSO-d_6)

Samples	δ_{HBD} (ppm)	δ_{HBA} (ppm)
Lactic acid	4.03	-
Citric acid	12.39	-
Malic acid	12.37	-
Maleic acid	6.30	-
Xylitol	-	3.51
Thymol	9.05	-
Menthol	-	4.29
DES-1	4.04	3.52
DES-2	12.40	3.54
DES-3	6.33	3.57
DES-4	12.37	3.52
DES-5	4.04, 12.51	3.54
DES-6	4.04, 6.36	3.54
DES-7	4.04, 12.49	3.55
DES-8	12.37	3.52
DES-9	6.37	3.54
DES-10	6.35	3.54
DES-11	4.06	4.32
DES-12	9.07	4.30

Supplemental Information

Table S2. Fitting results of the two models for DES cleaning of tea-stained bovine teeth

Kinetic models	DES concentration	Cleaning method	Kinetic constant	Kinetic equation	R^2
Ritger-Peppas	40%	Stirring	0.3770	y=0.3770x+2.0399	0.9194
		Electro-stirring	0.3318	y=0.3318x+2.2143	0.9654
		Ultrasonication	0.3388	y=0.3388x+2.2559	0.9577
Higuchi		Stirring	4.2102	y=4.2102x+0.2357	0.9044
		Electro-stirring	4.3453	y=4.3453x+0.3249	0.8991
		Ultrasonication	4.5907	y=4.5907x+0.3718	0.9135

Supplemental Information

Table S3. Fitting results of the two models for DES cleaning of coffee-stained bovine teeth

Kinetic models	DES concentrations	Cleaning methods	Kinetic constants	Kinetic equations	R^2
Ritger-Peppas	40%	Stirring	0.4622	y=0.4622x+1.6574	0.9176
		Electro-stirring	0.4441	y=0.4441x+1.7692	0.9089
		Ultrasonication	0.3967	y=0.3967x+2.0383	0.9075
Higuchi		Stirring	5.8995	y=5.8995x+0.7889	0.9534
		Electro-stirring	6.0842	y=6.0842x-0.1616	0.9530
		Ultrasonication	6.1277	y=6.1277x+2.5789	0.9266

Supplemental Information

Table S4. Parameters of DES-12 emulsion

DES concentrations	DES/water/surfactant	Cohesiveness (gf)	Viscosity (mPa·s)	Whiteness
5%	5:85:10	-9.31±0.36	178.98±4.15	45.41±0.41
20%	10:75:15	-12.17±0.49	241.77±4.97	84.25±0.31
40%	20:40:20	-12.41±0.43	954.67±8.89	95.96±0.54

Supplemental Information

Table S5. Inhibition zone sizes of 5% and 40% DES against *E. coli* and *S. aureus*.

Sample	DES concentration	*E. coli* (mm)	*S. aureus* (mm)
DES-3	5%	7.8±0.1	9.5±0.2
	40%	11.1±0.3	22.0±0.2
DES-5	5%	7.6±0.2	7.7±0.1
	40%	11.4±0.3	19.7±0.3
DES-12-P	5%	9.8±0.2	9.7±0.1
	40%	13.2±0.3	24.1±0.2

Supplemental Information

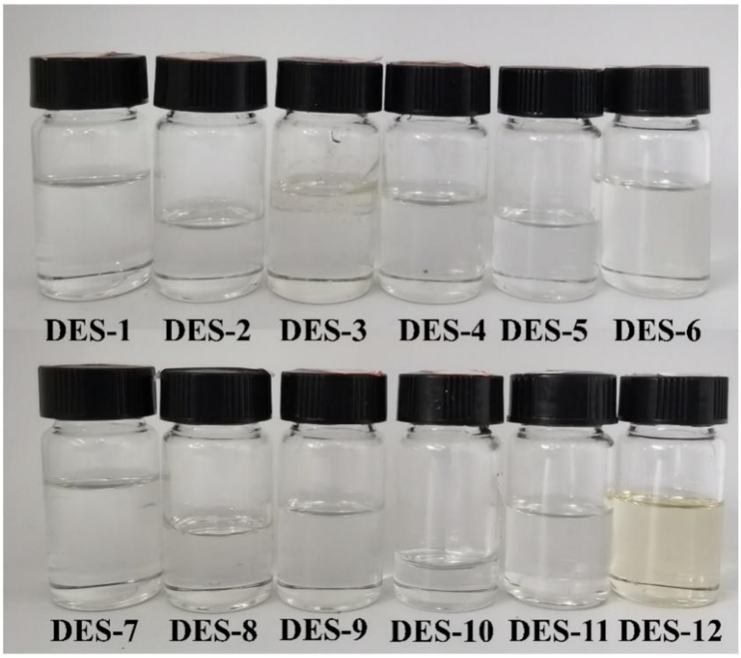

Fig. S1. Synthetic appearance of DESs

Supplemental Information

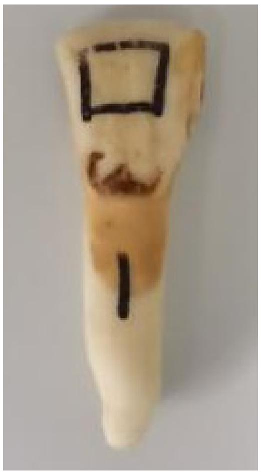

Fig. S2. Marked and stained bovine teeth sample

Supplemental Information

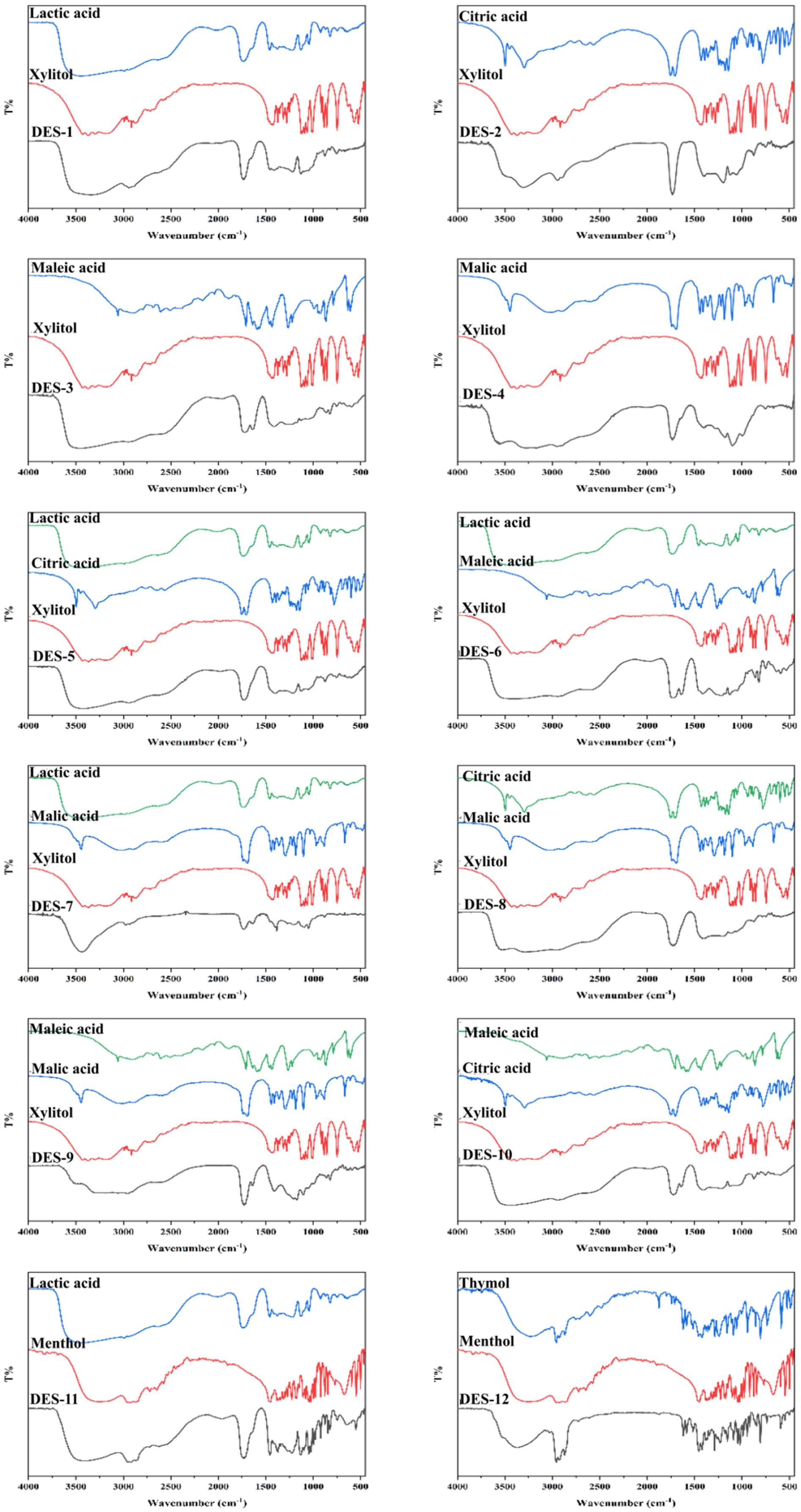

Fig. S3. Infrared spectra of DESs and their HBDs/HBAs (KBr disc)

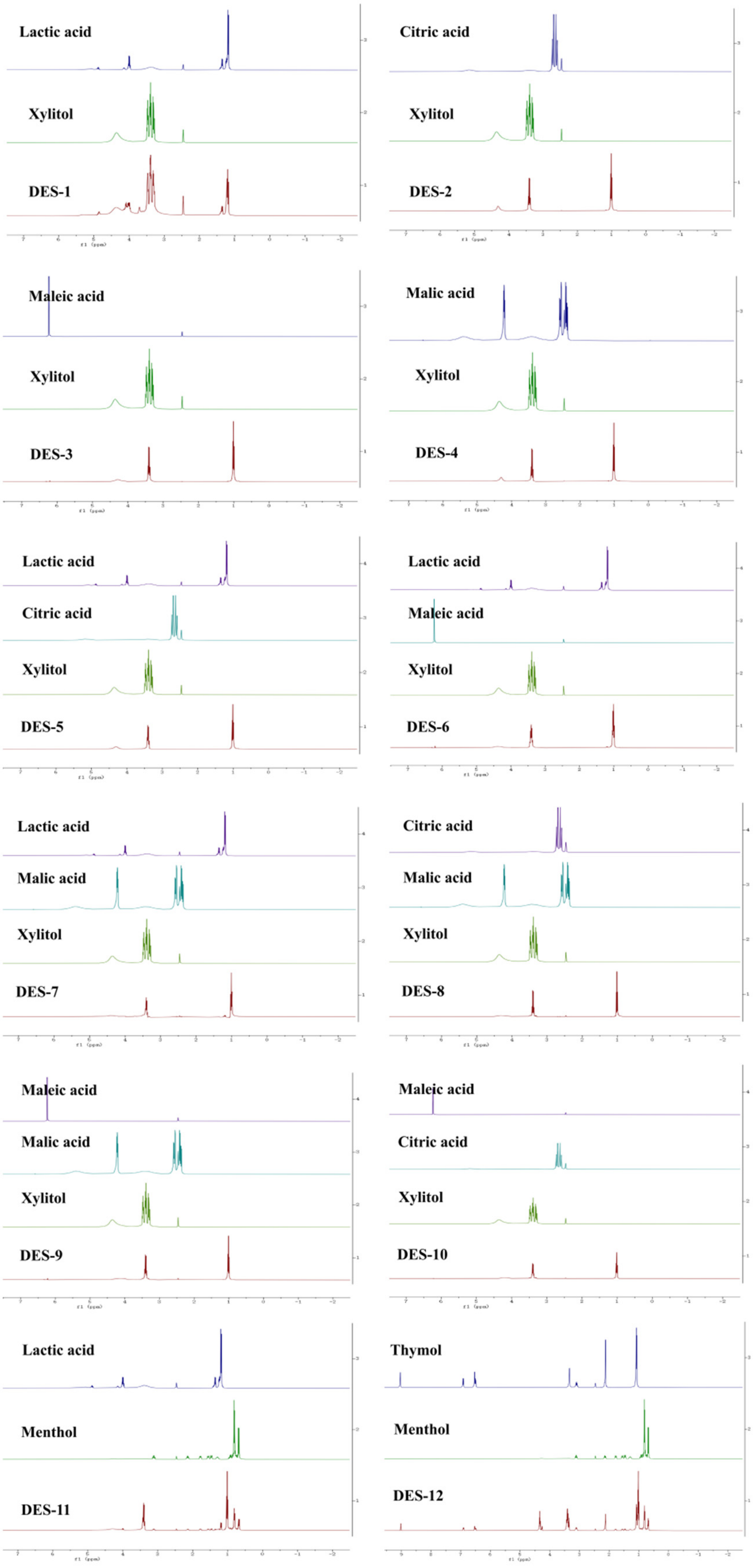

Fig. S4. ^{1}H NMR spectra of DES and their HBDs/HBAs (400 MHz, DMSO-d_6)

Supplemental Information

References

1. Zhang, Z.; Li, R.; Chen, Q.; Mahmood, S.; Yao, S. Foaming natural deep eutectic solvents (FNADES) based on natural sweeteners and its solubilization effect for food applications. *LWT-Food Sci. Technol.* **2025**, *234*, 118594, doi:10.1016/j.lwt.2025.118594.
2. Zhang, Z.; Mahmood, S.; Ali, A.; Ye, Z.; Yao, S. Fabrication of carbon dots from *Siraitia grosvenorii* shells and their fluorescent response for deep eutectic solvents. *Colloids and Surfaces a-Physicochemical and Engineering Aspects* **2025**, *721*, 137273, doi:10.1016/j.colsurfa.2025.137273.
3. Farias, F.O.; Bordon Sosa, F.H.; Igarashi-Mafra, L.; Pereira Coutinho, J.A.; Mafra, M.R. Study of the pseudo-ternary aqueous two-phase systems of deep eutectic solvent (choline chloride:sugars)+K_2HPO_4+water. *Fluid Phase Equilib.* **2017**, *448*, 143–151, doi:10.1016/j.fluid.2017.05.018.
